# *Listeria monocytogenes* Establishes Commensalism in Germ-Free Mice Through the Reversible Downregulation of Virulence Gene Expression

**DOI:** 10.3389/fimmu.2021.666088

**Published:** 2021-05-03

**Authors:** Kyungjin Cho, Darina Spasova, Sung-Wook Hong, Eunju O, Charles D. Surh, Sin-Hyeog Im, Kwang Soon Kim

**Affiliations:** ^1^ Department of Life Sciences, Pohang University of Science and Technology (POSTECH), Pohang, South Korea; ^2^ Division of Developmental Immunology, La Jolla Institute for Allergy and Immunology, La Jolla, CA, United States; ^3^ Division of Integrative Biosciences and Biotechnology, Pohang University of Science and Technology (POSTECH), Pohang, South Korea

**Keywords:** commensalism, *Listeria monocytogenes* (*L. monocytogenes*), germ-free mice, pathobionts, host-microbe interaction

## Abstract

The intestine harbors a complex community of bacterial species collectively known as commensal microbiota. Specific species of resident bacteria, as known as pathobiont, have pathogenic potential and can induce apparent damage to the host and intestinal inflammation in a certain condition. However, the host immune factors that permit its commensalism under steady state conditions are not clearly understood. Here, we studied the gut fitness of *Listeria monocytogenes* by using germ-free (GF) mice orally infected with this food-borne pathogen. *L. monocytogenes* persistently exists in the gut of GF mice without inducing chronic immunopathology. *L. monocytogenes* at the late phase of infection is not capable of infiltrating through the intestinal barrier. *L. monocytogenes* established the commensalism through the reversible down regulation of virulence gene expression. CD8^+^ T cells were found to be sufficient for the commensalism of *L. monocytogenes*. CD8^+^ T cells responding to *L. monocytogenes* contributed to the down-regulation of virulence gene expression. Our data provide important insights into the host-microbe interaction and have implications for developing therapeutics against immune disorders induced by intestinal pathogens or pathobionts.

## Introduction

Multicellular organisms have co-evolved with a complex community of microbial species, collectively known as the commensal microbiota (1). During the co-evolution of host-microbe interactions, environmental bacterial species exposed to the gastrointestinal tract establish commensalism, a state of infection that induces no or inapparent damage to the host, even though it can elicit immune responses (2). These commensal bacterial species can be either mutually beneficial or harmless to the host, and profoundly influence the host physiology in various aspects (3).

Certain intestinal pathogens can also adapt to occupy the niches in the intestine by inducing transient and limited pathogenesis. Gut fitness of pathogenic fungal or bacterial species can result from the evolutionary loss of virulence genes or from the reversible suppression of virulence gene expression in the gut (4–6). In the latter case, the bacterial species still possess a pathogenic potential, therefore termed pathobionts, and are involved in the pathogenesis of intestinal infection or inflammatory bowel diseases (7, 8). However, host-derived factors that promote commensalism of intestinal pathogens or pathobiont species are not fully understood due to the complexity of host-microbe interactions as well as inter-species interactions in the gut. Previously, it was reported that upon the oral infection in germ-free (GF) mice, *Citrobacter rodentium*, a murine intestinal pathogen that mimics attaching and effacing pathogens such as a pathogenic *Escherichia coli* in humans, can establish commensalism (5). Commensalism of *C. rodentium* in GF mice can be established by the immune-mediated clearance of virulent bacteria. IgG specific to virulence factors, which are encoded by the locus of enterocyte effacement (LEE), eliminates virulent bacteria in the gut (9). However, it is still unclear whether other intestinal pathogens can establish commensalism within the host and whether host-derived factors, other than antibodies, can promote commensalism.

*Listeria monocytogene*s is a typical food-borne and facultative Gram-positive pathogen that induces the listeriosis in humans (10). Upon the oral infection of *L. monocytogenes*, the surface protein, internalin A (InlA), of *L. monocytogenes* interacts with epithelial cadherin (E-cad) expressed on the small intestinal epithelial cells (IECs). This interaction leads to the traversal of *L. monocytogenes* through the small intestinal barrier and its subsequent spreading into internal organs (11). *L. monocytogenes* infection results in high mortality in immunocompromised individuals, pregnant women, neonates, and elderly individuals (12). However, in healthy individuals or in immunocompetent mice, *L. monocytogenes* only induces acute infection and transient symptoms due to the induction of protective immunity such as *L. monocytogenes*-specific CD8^+^ T cells and the presence of gut microbiota (13–15). Commensal gut microbiota contributes to the protection from *L. monocytogenes* infection; this phenomenon is called ‘colonization resistance’ (14, 16). In this regard, orally-infected *L. monocytogenes* disappears in the late phase of infection (14, 17). GF mice are more susceptible to *L. monocytogenes* infection. Higher bacteremia occurs in GF mice upon oral *L. monocytogenes* infection than in specific pathogen-free (SPF) mice (14, 18). However, *L. monocytogenes* can be carried asymptomatically in various animals as well as in humans (19), although the underlying mechanisms are not clearly understood. It remains elusive whether inter-species interactions between *L. monocytogenes* and other microbial species in the gut are required or whether the host-derived factors are sufficient for the asymptomatic carriage of *L. monocytogenes*.

In the present study, we investigated the long-term consequences of oral *L. monocytogenes* infection in GF mice and whether *L. monocytogenes* can transit from the pathogenicity to commensalism *in vivo*. By utilizing GF mice orally infected with *L. monocytogenes*, we showed that luminal *L. monocytogenes* can persist in the lumen of GF mice without inducing chronic immunopathology and establish the commensalism through the reversible downregulation of virulence gene expression. *L. monocytogenes*-specific CD8^+^ T cells are sufficient to promote the commensalism of luminal *L. monocytogenes* in GF mice by promoting the downregulation of certain virulence gene expression.

## Materials and Methods

### Mice

SPF C57BL/6 (B6), *Rag1^−/−^*, *JH* ^−/−^ and OT-I mice were purchased from Jackson Laboratory and maintained in the animal facility of POSTECH Biotech Center. GF B6 and *Rag1^−/−^* mice were kindly provided by Drs. A. Macpherson and K. McCoy (University of Bern, Switzerland) and maintained in sterile flexible film isolators (Class Biological Clean Ltd.) by feeding autoclaved Teklad global 18% protein rodent diets (2018S; Envigo, USA). Age-matched 9- to 12-week-old mice bred in our facility were used for all experiments. All animal experiments were conducted in accordance with the guidelines of the Institutional Animal Care and Use Committee of POSTECH.

### Oral Infection of *L. monocytogenes*

Recombinant *L. monocytogenes* strain S10403 genetically modified to express mutated internalin A (S192N and Y369S) and ovalbumin (designated as InlA^m^ *LM-OVA*) was kindly provided by Dr. B. Sheridan (University of Stony Brook). InlA^m^ *LM-OVA*, which is streptomycin resistant, was grown in Brain-Heart Infusion (BHI) broth (MC cell) containing streptomycin (200μg/ml) at 37°C**** with shaking at 220rpm for 2 hr. InlA^m^ *LM-OVA* was grown to OD600 at about 0.8, then washed with sterile PBS. Mice were infected by gavage with 0.2ml PBS containing 5 x 10^8^ CFU InlA^m^ *LM-OVA*.

### Lipopolysaccharide (LPS) Injection

To induce the expression of antimicrobial protein (AMP) such as *Reg3γ* in small intestinal epithelial cells (IECs), GF mice were intraperitoneally (i.p.) injected with LPS (20μg/mouse) prepared from Escherichia coli O26:B6 (Sigma-Aldrich, USA) for 3 consecutive days.

### Enumeration of *L. monocytogenes*

To enumerate bacterial burden in organs, collected organs were grinded in PBS containing 0.05% of Triton-X (DAE JUNG, Korea) with 100μm mesh cap. Grinded tissues were incubated at least 1h at 4°C****. To determine bacterial burden in feces, fecal contents were collected from individual mice in cold PBS. 10-fold serial dilutions of each sample were prepared in sterile PBS. 100μl of diluted samples was plated on BHI agar plate with 200μg/ml streptomycin and incubated overnight (18-24h) at 37°C****.

### Cell Isolation and Sorting

Single cell suspension from lymph nodes and spleen was prepared by mechanical dissociation through 100μm filter. For preparing single cell suspension from small lamina propria, small intestines were harvested and Peyer’s patches were removed. Small intestines were cut into small segment and incubated in PBS buffer containing 2% FBS, 20mM HEPES, 100U/ml penicillin and 100μg/ml streptomycin, 1mM sodium pyruvate and 20mM EDTA for 25min at 37°C to remove the epithelial cells and were washed extensively with PBS. Segments of tissues were digested with 400 Mandl units/ml Collagenase D (Roche) and 100U/ml DNase I (Roche) in RPMI medium containing 3% FBS, 20mM HEPES, 100U/ml penicillin and 100μg/ml streptomycin, 1mM sodium pyruvate and 1mM non-essential amino acid for 45min at 37°C with continuous stirring. Cell suspensions were enriched by 40:75% Percoll density gradient centrifuge. For isolation of small intestinal intraepithelial lymphocytes (IEL) and IECs, segments of small intestine were incubated in PBS buffer containing 2% FBS, 20mM HEPES, 100U/ml of penicillin and streptomycin, 1mM sodium pyruvate and 2mM EDTA for 25min at 37°C. After incubation, cell suspensions were enriched by 40:75% Percoll density for IEL and by 20:40% Percoll density for IEC. For IEC sorting, CD45^−^ Epcam^+^ IECs were FACS-sorted with Moflo XDP (Beckman Coulter, Brea, USA). The purity of sorted cells was routinely over 95%.

### Flow Cytometry

Isolated cells were washed with PBS and stained with Ghost viability dye (Tonbo) or propidium iodide to discriminate live from dead cells. For surface staining, cells were stained fluorochrome-labeled antibodies. CD8α (53-6.7), CD90.1 (Thy1.1, OX-7), CD127 (A7R34), KLRG1 (2Fa), IFN-γ (XMG1.2), and TNF-α (MP6-XT22) were purchased from Biolegend, Thermo Fisher Scientific, BD Biosciences. For intracellular cytokine staining, cells were stimulated for 4 hrs in RPMI-1640 medium containing 10% FBS, penicillin (100U/ml), streptomycin (100μg/ml), and 55μM β-mercaptoethanol in presence of OVA257-264 (SIINFEKL) peptide (PEPTRON) and Golgi plug (BD Biosciences), and surface-stained cells were fixed and permeabilized with Cytofix/Cytoperm kit (BD Biosciences). Stained cells were analyzed using LSRFortessa (BD Biosciences), and data were analyzed using Flowjo software (Tree Star).

### Repopulation of OT-I Cells

For adoptive transfer of OT-I cells, lymph node cells were harvested from Thy1.1^+^ OT-I *Rag1^−/−^* mice and 5 x 10^5^ cells were intravenously (i.v.) injected into recipient SPF and GF mice. To facilitate the repopulation of donor OT-I cells in the small intestine, OT-I transferred mice were gavaged with ovalbumin (OVA, Sigam-Aldrich, USA, grade V, 0.2mm filtered, 20mg/mouse) every other day for 10 days.

### *In Vivo* CD8^+^ T Cell Depletion

For depletion of donor OT-I T cells *in vivo*, OT-I-repopulated GF *Rag1^−/−^* mice were i.v. injected with 50μg of anti-CD8α antibody (clone YTS 169.4, BioXcell) per mouse every other day for 10 days.

### RNA Extraction and Quantitative Reverse-Transcription PCR (qRT-PCR)

For bacterial RNA preparation, the cecal contents of InlA^m^ *LM-OVA* infected GF B6 and *Rag1^−/−^* mice were harvested and bacterial RNAs were extracted by RNeasy PowerMicrobiome Kit (Qiagen, USA). For the preparation of RNA from FACS-sorted IECs, RNA was extracted by NucleoZOL (Macherey-Nagel, Germany). For cDNA preparation, genomic DNAs were first removed and cDNA synthesis were performed by Maxima first strand cDNA synthesis kit (Thermo Fisher Scientific). All processes were conducted according to the manufacturer’s instructions. qRT-PCR reactions were performed with PowerSYBRTM Green PCR Master Mix (Thermo Fisher Scientific) in total 10μl containing 1μl cDNA template, and 0.2μl of reverse and forward primer each (10μM). qRT-PCR reactions were run on Viia7 Real-time PCR system (Thermo Fishier Scientific) with the following cycles: 10min at 95****°C, and 40 cycles of 15 s at 95°C and 1min at 65°C. For AMP gene expression in IECs, signals were normalized to GAPDH transcript levels. For bacterial virulence gene expression in *L. monocytogenes*, signals were normalized to 16s rRNA transcript levels. Primers described in **Supplementary Tables 1** and **2** were used for the quantification of AMP and virulence gene expression by qRT-PCR, respectively (20–24).

### Immunofluorescence Staining and Confocal Microscopy

To examine the localization of *L. monocytogenes* in spleen and small intestine, tissues were embedded in optical cutting temperature compound (SAKURA Finetek, USA) and prepared as frozen blocks. Frozen sections were prepared at a 6-μm thickness by using a Leica CM1850 (Leica Microsystems, Germany). For foci of infection were visualized by staining with anti-*L. monocytogenes* antibody (ab35132; Abcam), followed by fluorescently labeled with Alexa-Flour 555 anti-rabbit IgG (H+L) (A32732; Invitrogen). Nuclei were visualized with 4’,6-diamidino-2-phenylindole (DAPI; Life Technologies). Image was acquired with Leica laser scanning confocal microscope (Leica Microsystems) equipped with 555-nm, and 632-nm laser channels.

### RNA Sequencing of IECs

RNA was extracted from FACS-sorted IECs using Trizol Reagent (Invitrogen, USA) by following the manufacturer’s procedure. Micro-RNA (mRNA) was isolated from total 1μg RNA by using oligodT. After removal of rRNA, mRNAs were reverse-transcribed to generate single-stranded cDNA using random hexamer and reverse transcriptase, followed by double-stranded cDNA synthesis. Double-stranded cDNA was fragmented to the appropriate size and used in a standard Illumina library preparation involving end-repair, A-tailing and adapter ligation, and PCR amplification. After quantification of library using KAPAlibrary quantification kit, RNA-sequencing library was sequenced on a NovaSeq (Illumina, USA) followed by cluster generation. Purified total RNA and RNA-sequencing were performed by Theragen Etex (Korea).

### Statistical Analysis

Mean ± S.E.M values were calculated by using Prism 8 (Graph Pad). Statistical significance was determined by unpaired two-tailed Student’s t test and one- or two-way ANOVA with Tukey’s multiple comparisons test. The Log-rank test was used to determine statistical differences in survival curves. *P*-values less than 0.05 were considered significant; *P < 0.05; **P < 0.01; ***P < 0.001.

## Results

### GF Mice Effectively Resolve Oral Infection of *L. monocytogenes* Despite Its Persistent Presence in the Lumen

To examine whether GF mice are capable of resolving oral infection of *L. monocytogenes*, we used recombinant *L. monocytogenes* genetically modified to express mutated internalin A (InlA^m^) and ovalbumin (OVA) as a surrogate antigen (designated as InlA^m^ *LM-OVA*) (15). Mutated InlA facilitates the interaction with murine E-cadherin. As described previously, InlA^m^ *LM-OVA* is highly effective in invading the small intestinal barrier in mice (15). We orally infected SPF and GF B6 mice with InlA^m^ *LM-OVA* at a dose of 5 x 10^8^ CFU/mouse.

**Figure 1:**
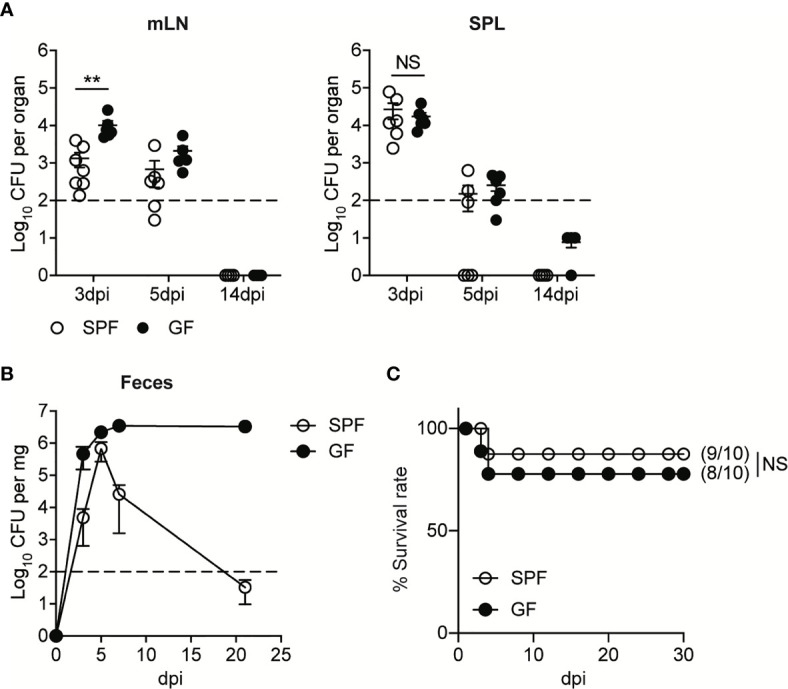
GF mice Effectively Resolve Oral Infection of *L. monocytogenes* Despite its Persistent Presence in the Lumen. SPF and GF mice were orally infected with 5 x 10^8^ CFU of InlA^m^ *LM-OVA.* **(A)** *L. monocytogenes* counts were determined as colony forming unit (CFU) in mesenteric lymph nodes (mLN) and spleen (SPL) of SPF and GF mice at indicated time points. Data point represents an individual mouse. Data are pooled from two independent experiments (n=6 at 3 and 5 dpi, n=4 at 14 dpi). **(B)** *L. monocytogenes* counts in feces were determined at the indicated time points. Data are representative of at least three independent experiments (n=4 per group). Statistical differences were determined by two-way ANOVA with Tukey’s multiple comparisons. **(C)** Survival rate at the indicated time points after oral infection of InlA^m^ *LM-OVA*. Numbers in parentheses indicate the ratio of surviving mice to total mice. The log-rank test was used to determine statistical differences in survival curves. Data are pooled from two independent experiments (n=10 per group). **P < 0.01; NS, not statistically significant.

Upon the oral infection, the kinetics of bacteremia in GF mice were similar to that of SPF mice. The levels of InlA^m^ *LM-OVA* in mesenteric lymph nodes (mLN) and spleen (SPL) reached a peak at 3 day post-infection (3 dpi) and then were gradually reduced below detection limit (**Figure 1A**). As shown previously (14, 18), GF mice were more susceptible to oral infection with InlA^m^ *LM-OVA* compared to SPF mice. GF mice had higher bacterial burdens in mesenteric lymph nodes (mLN) relative to SPF mice at 3 dpi despite comparable bacteremia in SPL (**Figure 1A**). Bacterial burdens in mLN and SPL at 14 dpi were reduced below the detection limit in both SPF and GF mice (**Figure 1A**). While luminal InlA^m^ *LM-OVA* was reduced below the detection limit at 21 dpi in SPF mice, InlA^m^ *LM-OVA* persistently existed at a high number in the lumen of GF mice (**Figure 1B**). Oral infection of InlA^m^ *LM-OVA* into GF mice was well-tolerated and the infected GF mice survived well (**Figure 1C**). These results suggest that GF mice are capable of eliminating tissue-infiltrating *L. monocytogenes* as efficiently as SPF mice despite the persistent presence of luminal *L. monocytogenes*.

### GF Mice Effectively Generate Systemic and Tissue-Resident Memory CD8^+^ T Cells Upon the Oral *L. monocytogenes* Infection.

CD8^+^ T cells are critical for the protection against *L. monocytogenes* infection and their clearance (13). *L. monocytogenes*-specific CD8^+^ T cells also effectively mediate the protection against secondary *L. monocytogenes* infection (15). To determine whether *L. monocytogenes*-specific CD8^+^ T cell responses are well established in GF mice, we examined *L. monocytogenes*-specific CD8^+^ T cell responses in SPF and GF mice after oral *L. monocytogenes* infection.

**Figure 2:**
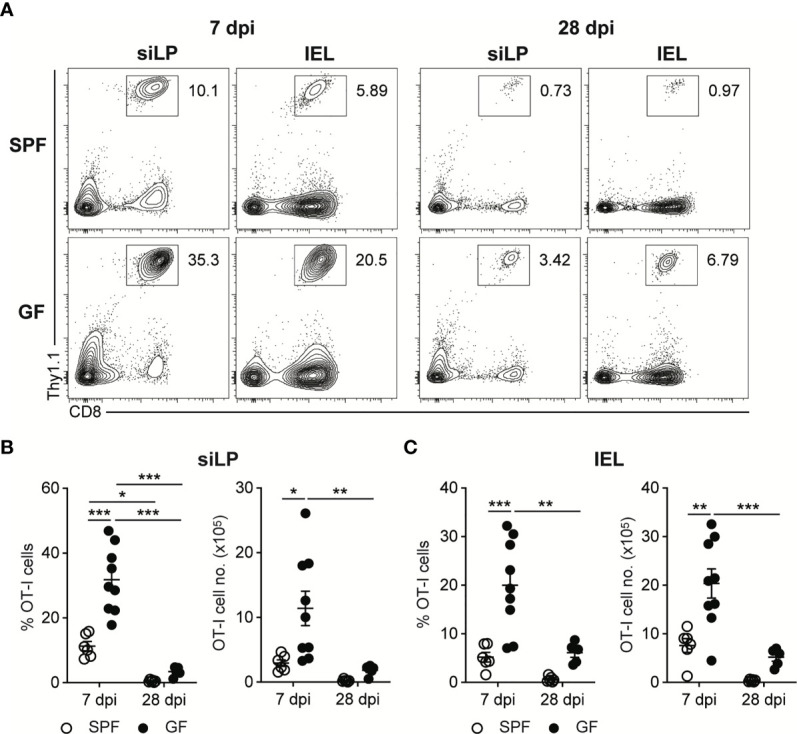
Oral *L. monocytogenes* Infection Generates Acute CD8^+^ T cells Response in GF mice as well as in SPF mice. 5 x 10^5^ OT-I cells were adoptively transferred into SPF and GF mice. Next day, mice were orally infected with InlA^m^ *LM-OVA*. Recovery of donor OT-I cells from small intestinal intraepithelial lymphocytes (IEL) and small intestinal lamina propria (siLP) was determined at 7 and 28 dpi. **(A)** Representative FACS plots showing the percentage of donor OT-I cells gated on lymphocytes. Percentage of donor OT-I cells (left) and total number of donor OT-I cells (right) in siLP **(B)** and IEL **(C)** at 7 and 28 dpi. Data point represents an individual mouse. Data are pooled from two independent experiments (n=6 for SPF 7 dpi, n=9 for SPF 28 dpi, n=9 for GF 7 dpi, n=5 for GF 28 dpi). Statistical differences were determined by two-way ANOVA with Tukey’s multiple comparisons. *P < 0.05, **P < 0.01, ***<0.001.

To follow up the CD8^+^ T cell responses against InlA^m^ *LM-OVA*, we adoptively transferred naïve OVA-specific CD8^+^ T cells (OT-I cells) into SPF and GF mice one day before oral infection. While the bacteremia of oral *L. monocytogenes* infection reached a peak at 3 dpi, OT-I cell responses reached a peak at 7 dpi in both SPF and GF mice (**Supplementary Figure S1A**). At the peak of CD8^+^ T cell responses, GF mice possessed a comparable number of OT-I cells in mLN and SPL relative to SPF mice (**Supplementary Figures S1B–D**). Meanwhile, the number of OT-I cells at 7 dpi was much higher in the small intestinal lamina propria (siLP) and small intestinal intraepithelial lymphocytes (IEL) in GF mice than in SPF mice (**Figures 2A–C**). Production of effector cytokines such as IFN-γ and TNF-α by donor OT-I cells in GF mice was comparable with that in SPF mice at 7 dpi (**Supplementary Figures 2A, B**).

In comparison to the number of donor OT-I cells at 7 dpi, the number of donor OT-I cells in both SPF and GF mice was markedly reduced, especially in intestinal tissues, at 28 dpi (**Figures 2A–C** and **Supplementary Figures S1A–C**). These results suggest that upon the oral infection with *L. monocytogenes*, GF mice effectively generate *L. monocytogenes*-specific CD8^+^ T cell responses especially in the intestinal tissues, and form systemic and intestinal tissue-resident memory CD8^+^ T cells after clearance of tissue-infiltrating *L. monocytogenes*.

### Luminal *L. monocytogenes* Fails to Infiltrate Through Intestinal Epithelium at the Late Phase of Infection in GF Mice

**Figure 3:**
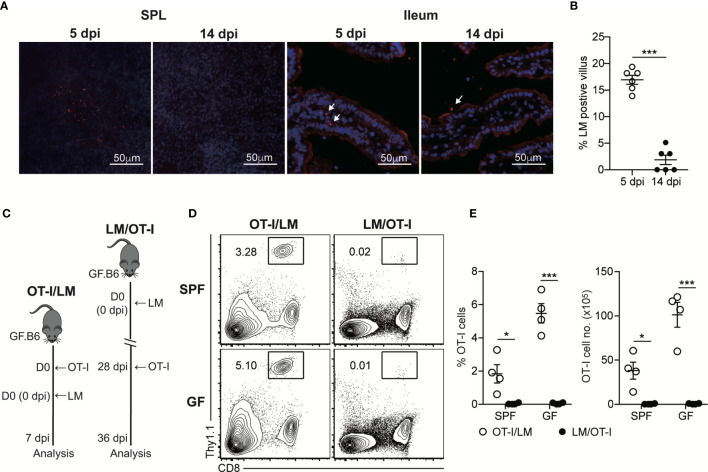
Luminal *L. monocytogenes* Fails to Infiltrate Through the Intestinal Epithelium at the Late Phase of Infection in GF mice. **(A)** GF mice were orally infected with InlA^m^ *LM-OVA*. SPL and ileum tissues were prepared at 5 and 14 dpi, and stained for *L. monocytogenes* (red) and nucleus (blue). Arrows indicate the foci of *L. monocytogenes* in siLP or lumen. **(B)** Percentage of villus containing *L. monocytogenes* in ileum at 5 and 14 dpi (n=6 per group). **(C-E)** 5 x 10^5^ OT-I cells were adoptively transferred into naïve SPF and GF mice and infected with InlA^m^ *LM-OVA* next day (OT-I/LM). 5 x 10^5^ OT-I cells were adoptively transferred into SPF and GF mice previously orally-infected with InlA^m^ *LM-OVA* at 28dpi (LM/OT-I). **(C)** Scheme of the experiment. **(D)** Representative FACS plots showing the percentage of OT-I cells gated on lymphocytes in SPL at day 8 after adoptive transfer. **(E)** Percentage of OT-I cells gated on live lymphocytes (left) and total numbers (right) of OT-I cells in SPL from indicated mice. Data are representative of two independent experiments (n=3~4 per group). Statistical differences were determined by unpaired two-tailed Student’s t test or two-way ANOVA with Tukey’s multiple comparisons. *P < 0.05, ***P < 0.001.

Our findings suggested that luminal *L. monocytogenes* persistently present in GF mice at the late phase of infection was unable to induce chronic CD8^+^ T cell activation, even in the intestinal tissues. To determine whether luminal *L. monocytogenes* fails to infiltrate through the intestinal barrier at the late phase of infection, we examined the localization of *L. monocytogenes* during the oral infection by confocal microscopic analysis. InlA^m^ *LM-OVA* at the early phase of infection (5 dpi) was capable of infiltrating the intestinal barrier and could be detected in SPL and also at the siLP underneath the epithelial barrier (**Figure 3A**). In contrast, even at 14 dpi, the number of InlA^m^ *LM-OVA* localized inside the siLP was significantly reduced although its localization was in a proximity to the intestinal epithelium. At 5 dpi, the percentage of villus that contained InlA^m^ *LM-OVA* was approximately 16% while the percentage of *L. monocytogenes*-positive villi was less than 5% at 14 dpi (**Figure 3B**).

To further examine the failure of tissue infiltration of luminal *L. monocytogenes*, we adoptively transferred OT-I cells into SPF and GF mice previously infected with InlA^m^ *LM-OVA* 21 days before the transfer (LM/OT-I). As a control, OT-I cells were adoptively transferred to SPF and GF mice, which were then orally infected with InlA^m^ *LM-OVA* (OT-I/LM) (**Figure 3C**). Oral infection of InlA^m^ *LM-OVA* into both SPF and GF mice reconstituted with OT-I cells (OT-I/LM) resulted in a prominent increase in donor OT-I cells (**Figures 3D, E**). As expected from the clearance of InlA^m^ *LM-OVA* in the lumen of SPF mice at 21 dpi, OT-I cells transferred into SPF mice previously infected with InlA^m^ *LM-OVA* failed to proliferate (**Figures 3D, E**). Interestingly, despite the persistent presence of InlA^m^ *LM-OVA* in the lumen of GF mice, luminal InlA^m^ *LM-OVA* in GF mice at the late phase of infection did not induce the effective proliferation of donor OT-I cells (**Figures 3D, E**). These results suggest that during the late phase of infection, *L. monocytogenes* in the lumen of GF mice is unable to infiltrate through the intestinal barrier and *L. monocytogenes* is capable of establishing commensalism within the host.

### Oral Infection of *L. monocytogenes* Induces the Prolonged Upregulation of Antimicrobial Protein Expression in Intestinal Epithelial Cells

The above findings indicate that oral infection of *L. monocytogenes* into GF mice only induces acute CD8^+^ T cell responses, although *L. monocytogenes* is persistently present in the lumen. Next, we examined innate immunity induced by oral infection of *L. monocytogenes* in GF mice. Intestinal epithelial cells (IECs) play an important role in the protection against intestinal pathogens by providing physical and chemical barrier functions (25, 26). IECs or Paneth cells directly respond to bacterial molecules from commensal gut microbiota and produce antimicrobial proteins (AMPs) that confer colonization resistance to intestinal pathogens (16, 27). To elucidate whether *L. monocytogenes* contributes to the intestinal barrier functions mediated by AMPs as an effector arm of innate immunity, we examined the alteration of gene expression programs in IECs upon the oral infection with *L. monocytogenes* in GF mice by RNA sequencing analyses on FACS-sorted CD45^−^ Epcam^+^ IECs. We selected two time points, 5 dpi *vs*. 14 dpi, for the analysis since tissue-infiltrating *L. monocytogenes* were obviously detected in mLN and siLP at 5 dpi but not at 14 dpi (**Figures 1A** and **3A, B**).

**Figure 4:**
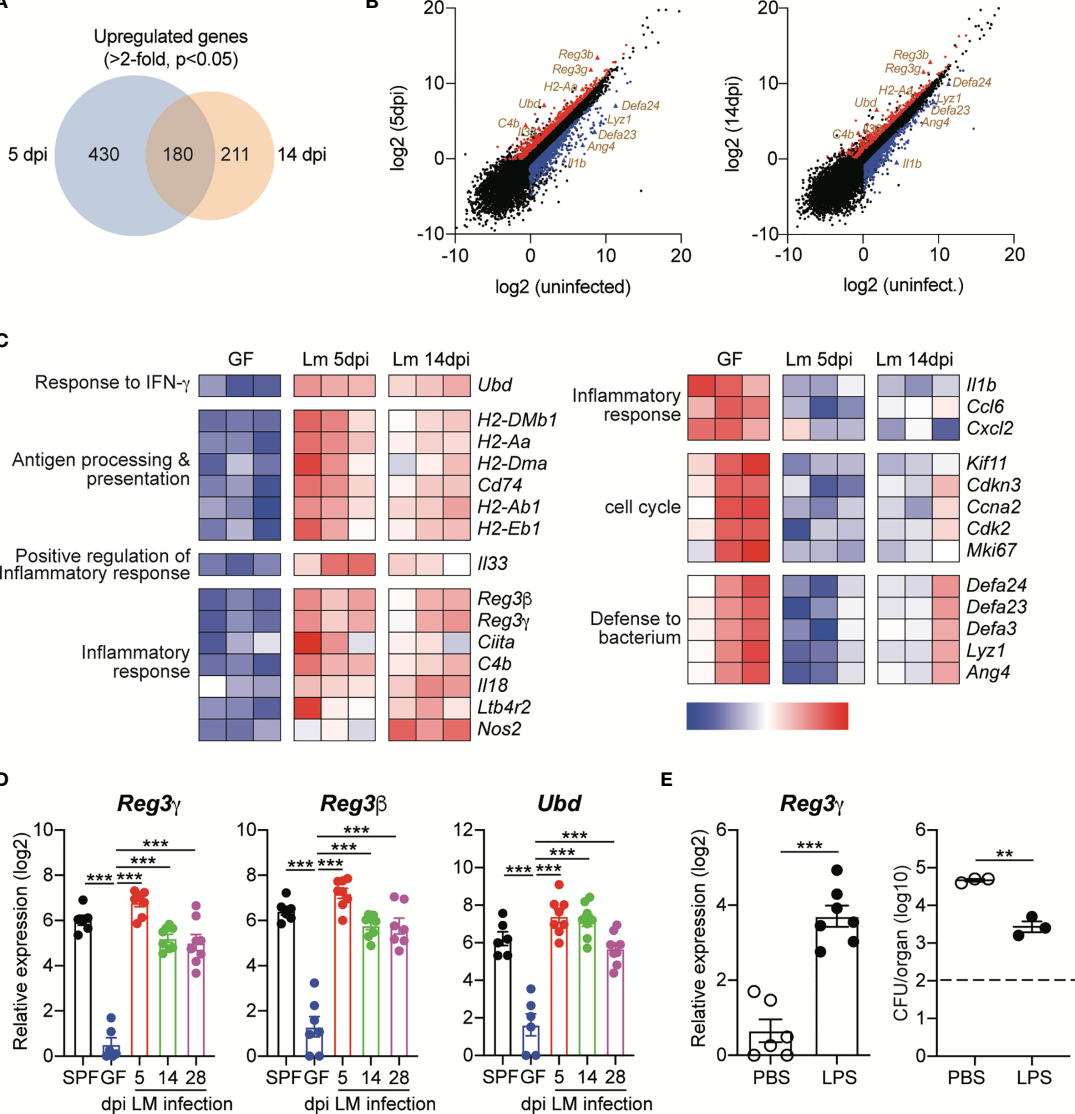
Oral Infection with *L. monocytogenes* Induces Prolonged Upregulation of Antimicrobial Protein Expression in Intestinal Epithelial Cells. Small intestinal epithelial cells (IECs) from GF mice were collect at indicated time points after InlA^m^ *LM-OVA* infection and then subjected to RNA-seq gene expression analysis. Differentially expressed genes (DEGs) were identified using three independent comparisons; uninfected *vs*. 5dpi, uninfected *vs*. 14dpi, and 5dpi *vs*. 14dpi. **(A)** The Venn diagrams showing the overlap between upregulated genes in IECs from infected GF mice at 5 dpi and 14 dpi relative to gene expression in IECs from uninfected GF mice. **(B)** Volcano plot of RNA-seq transcriptome data displaying the pattern of gene expression values for IECs at 5 dpi and 14 dpi *vs*. uninfected IECs. The differentially expressed genes (FDR < 0.05 and log2 fold change > 1) are shown. Upregulated or downregulated genes are indicated by red or blue color, respectively. **(C)** Heat map showing detailed expression information in IECs from uninfected GF, GF infected with InlA^m^ *LM-OVA* at 5dpi and 14dpi. Each column represents an individual mouse (n=3 per group). **(D)** Total RNA was prepared from IECs of uninfected SPF and GF mice, and InlA^m^ *LM*-OVA infected GF mice at indicated time points. Expression of *Reg3g*, *Reg3b*, and *Ubd* was measured by qRT-PCR. Data are pooled from two independent experiments (n=6~8 per group). **(E)** GF mice were i.p. injected with PBS or LPS (20μg per mouse) for 3 consecutive days and then infected with InlA^m^ *LM*-OVA. At 5 dpi, IECs were sorted to examine the expression of *Reg3g* gene (n=6 per group) and to measure tissue-infiltrating bacteria in mLN (n=3 per group). *Reg3g* gene expression (left) and bacteria loads in mLN (right). Statistical differences were determined by one-way ANOVA with Tukey’s multiple comparisons or unpaired two-tailed Student’s t test. **P < 0.01, ***<0.001.

Relative to those from uninfected GF mice, 630 and 391 genes were upregulated (≥2-fold with a false discovery rate-adjusted *P* value cutoff of 0.05) in IECs from infected GF mice at 5 dpi and 14 dpi, respectively (**Figure 4A**). Among them, 180 genes were commonly upregulated in IECs at both time points, including *H2-Aa, C4b, Reg3g, Reg3b* and *Ubd* (**Figures 4B, C**). The proteins encoded by the latter three genes are known to mediate the protection of intestinal pathogens (23, 28, 29). Regenerating islet-derived protein 3γ (Reg3γ) is a bactericidal lectin that is secreted into the lumen and preferentially binds to *L. monocytogenes* (30). Reg3γ production is increased by oral *L. monocytogenes* infection, thereby contributing to the protection against the infection (28). Reg3β and ubiquitin D (Ubd, also known as FAT10 protein) are known to mediate protection against *Salmonella* Typhimurium infection (23, 29).

We also verified the upregulation of these genes in IECs from GF mice infected with InlA^m^ *LM-OVA* by quantitative reverse transcription PCR (qRT-PCR) (**Figure 4D**). Interestingly, relative to that in uninfected GF mice, the expression of these genes was persistently upregulated in GF mice infected with InlA^m^ *LM-OVA*. Even at the late phase of infection (28 dpi), *Reg3g*, *Reg3b* and *Ubd* were significantly increased relative to those in uninfected GF mice, and the expression levels of these genes were comparable with those in SPF mice (**Figure 4D**).

To further examine the role of AMPs in the prevention of tissue infiltration of *L. monocytogenes*, GF mice were intraperitoneally injected with lipopolysaccharide (LPS) before oral infection with *L. monocytogenes*. IECs responded to LPS through TLR4 signaling and induced *Reg3g* expression (31). As expected, LPS injection led to the upregulation of *Reg3g* in IECs (**Figure 4E**). LPS treatment significantly reduced, but did not completely prevent, tissue infiltration of *L. monocytogenes* relative to that of PBS-treated GF mice (**Figure 4E**). Overall, these results suggest that in contrast to acute CD8^+^ T cell responses against *L. monocytogenes*, oral infection with *L. monocytogenes* induces the upregulation of AMP expression by IECs for a prolonged period, even though the commensalism of *L. monocytogenes* is established.

### Luminal *L. monocytogenes* in GF Mice Establishes Commensalism Through the Downregulation of Virulence Gene Expression

It was previously reported that the opportunistic fungal pathogen, *Candida albicans* can evolve in the gut of mice to lose their virulence genes in their genome through the long-term colonization and serial passage into uninfected host (4). Although GF mice were colonized with InlA^m^ *LM-OVA* for a short period (less than one month) without serial passages to uninfected GF mice, it is possible that the loss of the virulence gene program for the evolutionary fitness of *L. monocytogenes* in the gut can be responsible for its inability to infiltrate the intestinal barrier.

**Figure 5:**
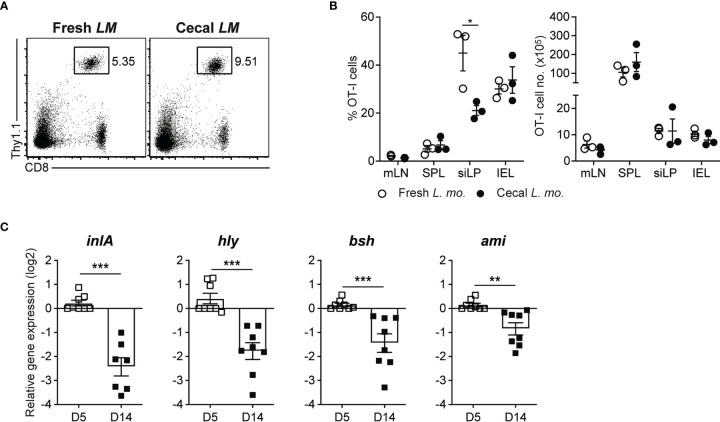
Luminal *L. monocytogenes* in GF mice Establish Commensalism Through the Downregulation of Virulence Gene Expression. **(A, B)** 5 x 10^5^ OT-I cells were adoptively transferred into GF mice. Next day, mice were orally infected with either InlA^m^ *LM-OVA* freshly prepared *in vitro* (Fresh LM) or InlA^m^ *LM-OVA* isolated from cecum of GF mice previously infected with InlA^m^ *LM-OVA* (Cecal LM). **(A)** Representative FACS plots showing the percentage of OT-I cells gated on lymphocytes in SPL at 7 dpi. **(B)** Percentage of OT-I cells gated on lymphocytes (left) and total numbers (right) of OT-I cells in indicated tissues from InlA^m^ *LM-OVA* infected GF mice at 7 dpi. Data are representative of two independent experiments (n=3 per group). Statistical differences were determined two-way ANOVA with Tukey’s multiple comparisons. *P < 0.05. **(C)** qRT-PCR analysis for the expression of the virulence genes (*inlA*, *hly*, *bsh*, and *ami*) in InlA^m^ *LM-OVA* isolated from InlA^m^ *LM-OVA* infected GF mice at 5 and 14 dpi. Gene expression was normalized to bacterial 16s RNA. Results are pooled from at least two independent experiments (n=8 per group). Statistical differences were determined by unpaired two-tailed Student’s t test. **P < 0.01, ***P < 0.001.

To address this issue, we adoptively transferred OT-I cells into GF mice and then infected them with InlA^m^ *LM-OVA*, which was harvested from the cecum of GF mice previously infected with InlA^m^ *LM-OVA* for 30 days. As a control, OT-I-reconstituted GF mice were infected with freshly *in vitro*-cultured InlA^m^ *LM-OVA*. Infection of cecal InlA^m^ *LM-OVA* led to the vigorous proliferation of OT-I cells as efficiently as that of InlA^m^ *LM-OVA* freshly prepared *in vitro*. These results indicate that luminal InlA^m^ *LM-OVA* at the late phase of infection did not lose their pathogenic capacity permanently, as seen in *C. albicans* infection (**Figures 5A, B**).

Next, to elucidate whether the reduction in virulence gene expression occurs during oral *L. monocytogenes* infection in GF mice, as seen in *C. rodentium* infection (5, 9), we examined the gene expression of well-defined virulence factors such as *InlA, Hly, Bsh*, and *Ami* in luminal InlA^m^ *LM-OVA* in GF mice by qRT-PCR. We found that the expression of these genes was profoundly downregulated at 14 dpi relative to that in luminal InlA^m^ *LM-OVA* at 5 dpi (**Figure 5C**). Collectively, these results suggest that reversible downregulation of virulence factors in luminal *L. monocytogenes*, but not the evolutionary loss of virulence factor genes, is responsible for the commensalism of *L. monocytogenes* in GF mice.

### *L. monocytogenes*-Specific CD8^+^ T Cells Are Sufficient to Facilitate the Commensalism of Orally-Infected *L. monocytogenes*

**Figure 6:**
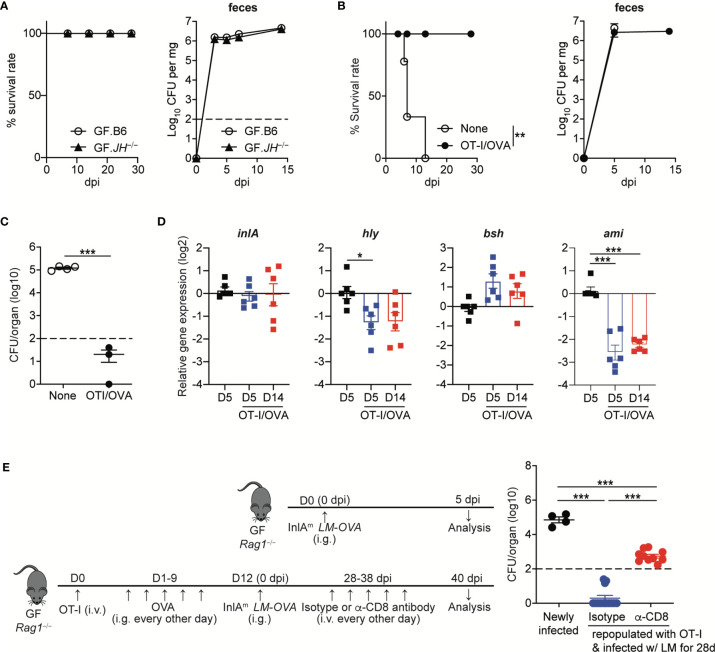
*Listeria*-specific CD8^+^ T Cells are Sufficient to Facilitate the Commensalism of Orally-infected *L. monocytogenes*. **(A)** GF *JH−/−* and B6 mice were orally infected with InlA^m^ *LM-OVA*. Survival rate (left) and the bacterial loads in feces (right) were determined at the indicated time points (for survival, n=10 per group; for bacterial loads in feces, n=4 per group). (B to D) 5 x 10^5^ OT-I cells were adoptively transferred into GF *Rag1^−/−^* mice, which were then fed with OVA (20mg/mouse) every other day by gavage for 10 days. GF *Rag1^−/−^* mice (None) and OT-I-repopulated GF *Rag1^−/−^* mice (OT-I/OVA) were infected with InlA^m^ *LM-OVA*. **(B)** Survival rate (left) and the bacterial loads in feces (right) (for survival, n=10 per group; for bacterial loads in feces, n=4 per group). **(C)** Bacterial loads were determined in the mLN from GF *Rag1^−/−^* mice (None) and OT-I-repopulated GF *Rag1^−/−^* mice (OT-I/OVA) at 5 dpi. Data are representative of two independent experiments (n=3~4 per group). The log-rank test was used to determine statistical differences in survival curves (A and B). **P < 0.01. **(D)** Expression of *inlA*, *hly*, *bsh*, and *ami* genes in InlA^m^ *LM-OVA* from the indicated GF *Rag1^−/−^* mice was measured by qRT-PCR. Gene expression was normalized to bacterial 16s RNA. Data are pooled from two independent experiments (n=6 per group). **(E)** OT-I-repopulated GF *Rag1^−/−^* mice were i.v. treated with PBS or anti-CD8α antibody every other days for 10 days to deplete reconstituted OT-I cells. As a control, GF *Rag1^−/−^* mice were orally infected with InlA^m^ *LM-OVA*. Bacterial loads were determined in the mLN at day 3 after the final anti-CD8α antibody treatment. Experimental scheme (left) and bacterial loads (right). Data are pooled from two independent experiments (n=4 for newly infected GF *Rag1^−/−^* mice, n=10 per OT-I-repopulated GF *Rag1^−/−^* mice infected with InlA^m^ *LM-OVA* and then treated with isotype or anti-CD8α antibody). Statistical differences were determined by unpaired two-tailed Student’s t test or one-way ANOVA with Tukey’s multiple comparisons. *P < 0.05, ***P < 0.001.

As shown in *C. rodentium* infection (9), it is plausible that virulence factor-specific antibody responses are necessary for the commensalism of *L. monocytogenes*. Therefore, we first examined whether B cells are required for the commensalism of *L. monocytogenes*. We orally infected GF B cell-deficient *JH^−/−^* mice with InlA^m^ *LM-OVA* at a dose of 5 x 10^8^ CFU/mouse. The GF *JH^−/−^* mice tolerated the oral infection well and survived well until the end of the experiment (over one month) (**Figure 6A**). InlA^m^ *LM-OVA* reached a plateau in numbers with kinetics similar to that seen in GF B6 mice (**Figure 6A**). Hence, in contrast to the critical role of IgG responses in the commensalism of *C. rodentium*, B cells are dispensable for the commensalism of *L. monocytogenes* in GF mice.

Luminal *L. monocytogenes* established commensalism between 5 dpi and 14 dpi as judged from the bacteremia in mLN and SPL, and the levels of *L. monocytogenes* traversing through intestinal barrier (**Figures 1A** and **3A, B**). Furthermore, the peak of *L. monocytogenes*-specific CD8^+^ T cell responses could be detected during this interval. In this regard, we investigated whether CD8^+^ T cells can promote the commensalism of *L. monocytogenes*. GF *Rag1^−/−^* mice lacking B and T cells were infected with InlA^m^ *LM-OVA*. OT-I cells were adoptively transferred into one cohort of GF *Rag1^−/−^* mice, which were fed with OVA to promote the repopulation of OT-I cells in the intestine (designated as OT-I/OVA).

GF *Rag1^−/−^* mice were highly vulnerable to InlA^m^ *LM-OVA* infection and all infected GF *Rag1^−/−^* mice succumbed to death within 2 weeks (**Figure 6B**). However, GF *Rag1^−/−^* mice repopulated with donor OT-I cells survived well, although luminal bacteria burdens in OT-I-repopulated GF *Rag1^−/−^* mice were similar to those in GF *Rag1^−/−^* mice (**Figure 6B**). Repopulation of OT-I cells effectively reduced the level of tissue-infiltrating InlA^m^ *LM-OVA* in mLN at 5 dpi (**Figure 6C**).

Interestingly, in contrast to the marked increase in AMP expression in IECs from GF B6 mice infected with InlA^m^ *LM-OVA*, GF *Rag1^−/−^* mice orally infected with InlA^m^ *LM-OVA* did not display a marked increase in AMP expressions in IECs (**Supplementary Figure S3**). Repopulation of donor OT-I cells facilitated the prominent reduction of *ami* gene expression and, to a lesser extent, *hly* gene expression, but not *inlA* and *bsh* gene expression, even at 5 dpi (**Figure 6D**). *In vivo* depletion of OT-I cells in OT-I-repopulated GF *Rag1^−/−^* mice by the treatment with anti-CD8α antibody resulted in the increased tissue infiltration of InlA^m^ *LM-OVA* in mLN, albeit at lower levels than that in GF *Rag1^−/−^* mice newly infected with InlA^m^ *LM-OVA* (**Figure 6E**). These results suggest that *L. monocytogenes*-specific CD8^+^ T cells are sufficient to permit the commensalism of *L. monocytogenes* in GF mice and contribute to the downregulation of certain virulence gene expression.

## Discussion

In this study, we demonstrated that *L. monocytogenes*, an obligated food-borne pathogen, can establish the commensalism in GF mice due to the reversible downregulation of virulence gene expression, but not due to the evolutionary loss of its virulence genes. Commensalism of *L. monocytogenes* in GF mice is supported by the lack of tissue infiltration at the late phase of infection, no apparent mortality, and the induction of innate immunity, such as AMP expression by IECs, although *L. monocytogenes* at the late phase of infection is not capable of inducing adaptive immunity. These findings represent the adaptation of environmental pathogenic species to become a member of the commensal gut microbiota, in particular pathobiont species, by tightly regulating its virulence gene expression. To our knowledge, this is the first study to show that in addition to antibodies against virulence factors, CD8^+^ T cells can be a key host factor that promotes the commensalism of intestinal pathogens.

Upon the oral infection with *L. monocytogenes*, GF B6 and *Rag1^−/−^* mice repopulated with OT-I cells effectively induced the clearance of tissue-infiltrating *L. monocytogenes*, prevented mortality, and permitted the commensalism of *L. monocytogenes*. However, the underlying mechanisms of the protection from *L. monocytogenes* and the establishment of commensalism can differ depending on the host. Upon the oral infection with *L. monocytogenes*, the expression of AMPs by IECs was prominently upregulated and maintained at high levels in GF B6 mice, but not in GF *Rag1^−/−^* mice repopulated with OT-I cells. Further studies are required to explain the differential regulation of AMP gene expression in IECs depending on the host. Nevertheless, as the production of AMPs by IECs provides the protection against oral *L. monocytogenes* infection, AMP production by IECs might compensate for the reduced number of *L. monocytogenes*-specific CD8^+^ T cells at the late phase of infection to prevent the tissue infiltration of *L. monocytogenes* in GF B6 mice. Our experimental data on OT-I-repopulated *Rag1^−/−^* mice suggest that even in the absence of marked AMP expression, CD8^+^ T cells sufficiently promote commensalism of luminal *L. monocytogenes*.

Repopulation of OT-I cells into GF *Rag1^−/−^* mice can promote the downregulation of virulence factor genes, particularly, the *ami* gene, which encodes an autolysin, upon the oral infection with InlA^m^ *LM-OVA*. Autolysin mediates adherence of *L. monocytogenes* to IECs (32). Rapid reduction of virulence gene expression in luminal *L. monocytogenes* in OT-I repopulated GF *Rag1^−/−^* mice might be caused by the presence of activated OT-I cells, which were generated by OVA-feeding. Interestingly, the pattern of virulence gene expression in *L. monocytogenes* from OT-I-repopulated GF *Rag1^−/−^* mice was different from that in GF B6 mice, suggesting that additional host factors other than CD8^+^ T cells might promote the downregulation of multiple virulence gene expression. Although B cells are dispensable for the commensalism of *L. monocytogenes* in GF mice, antibodies to virulence factors on the cell surface of *L. monocytogenes* can contribute to its commensalism presumably by the neutralization of virulence factors that mediate adherence to epithelial cells and tissue invasion (33) or alternatively by the elimination of virulent *L. monocytogenes* as seen in *C. rodentium* infection (9).

It is still unclear how CD8^+^ T cells can promote the commensalism of luminal *L. monocytogenes* in GF mice. Downregulation of *ami* gene expression might not be sufficient to prevent the invasion of *L. monocytogenes* through the intestinal barrier. Previously, it was reported that granzymes, a serine protease produced by CD8^+^ T cells, can target multiple proteins in biosynthetic and metabolic pathways of microbial pathogens (34). Therefore, it is possible that granzymes produced by intestinal CD8^+^ T cells and secreted into the lumen can target signaling pathways required for the expression of virulence factors at the transcriptional level. Furthermore, it was recently reported that granzymes can cleave virulence factors of *L. monocytogenes* (35). In this regard, granzymes produced by intestinal CD8^+^ T cells can result in the degradation of virulence factors in luminal *L. monocytogenes*, thereby further limiting the virulence of luminal *L. monocytogenes*.

Fecal microbiota transplantation (FMT) has been proven to be highly effective in the treatment of recurrent *Clostridioides difficile* infection (36). However, the safety concerns regarding FMT have been raised since a patient had died after receiving FMT. A study revealed that the invasive infection of multi-drug resistant *Escherichia coli*, which are indigenous in fecal preparation from healthy donors, was responsible for the death of a patient who underwent FMT (37). As exemplified in the present study, the identification of such pathobiont species and an understanding of the mechanisms that promote its commensalism are necessary for developing safe and effective microbiome-based therapeutics such as FMT. Our data provide an important insight into the host-microbe interactions that contribute to the commensalism of intestinal pathogens and have implications in developing therapeutics against immune disorders induced by intestinal pathogens.

## Data Availability Statement

RNA-seq data were deposited into the Sequence Read Archive (SRA) database (http://ncbi.nlm.nih.gov/sra) (accession number for SRA data: PRJNA693433).

## Ethics Statement

The animal study was reviewed and approved by POSTECH Institutional Biosafety Committee (PIBC-033).

## Author Contributions

CD and KK conceived the study. KC, CD, and KK participated in experimental designs for this study. KC, DS, S-WH, and EO conducted the experiments, acquired and analyzed the data. KC, S-HI and KK wrote the paper and prepared the figures. All authors contributed to the article and approved the submitted version.

## Funding

This work was supported by the National Research Foundation of Korea (NRF) grant (No. NRF-2020R1A2C1008459) and grant from Institute for Basic Science (IBS-R0005-D1) both funded by the Korea government (MSIT).

## Conflict of Interest

S-HI is the CEO of the ImmunoBiome, but declares no conflicts of interest for this research.

The remaining authors declare that the research was conducted in the absence of any commercial or financial relationships that could be construed as a potential conflict of interest.

## Abbreviations

AMP, Antimicrobial protein; *C. albican*, *candida albican*; *C. rodentium*, *Citrobacter rodentium*; day post-infection, dpi; FMT, fecal microbiota transplantation; GF, germ-free; LPS, lipopolysaccharide; *L. monocytogenes*, *Listeria monocytogenes*; mLN, mesenteric lymph node; mRNA, micro-RNA; Reg3γ, regenerating islet-derived protein 3γ; IEL, intraepithelial lymphocyte; siLP, small intestinal lamina propria; SPF, specific pathogen-free; SPL, spleen.
