# Supplementary material for: Listeria monocytogenes Establishes Commensalism in Germ-Free Mice Through the Reversible Downregulation of Virulence Gene Expression
Source: Front Immunol. 2021 May 3;12:666088. doi: 10.3389/fimmu.2021.666088 (PMC8126713; doi:10.3389/fimmu.2021.666088)
Supplement: Supplementary file 1 [file DataSheet_1.pdf]

Table S1. Oligonucleotides used in qRT-PCR assays for virulence gene expression in *L. monocytogenes*

Target gene	oligonucleotide sequences (5'-3')	
	Forward	Reverse
16s RNA	CTTCCGCAATGGACGAAAGT	ACGATCCGAAAACCTTCTTCATAC
inlA	GAACCAGCTAAGCCIGTAAAAG	CGCCIGTTTGGGCATCA
hly	TGCAAGTCCTAAGACGCCA	CACTGCATCTCCGTGGTATACTAA
bsh	AGCCAATTCCGCCCATCCCAC	CGCGTTCTTTCGAGTGAAACCCCA
ami	GGACTGCAACGGAAGAACATG	CTGTTGTTGCGCGAAGCA

Table S2. Oligonucleotides used in qRT-PCR assays for AMP gene expression in IECs

Target gene	oligonucleotide sequences (5'-3')	
	Forward	Reverse
GAPDH	CATGGCCTTCCGTGTTCCTA	CCTGCTTCACCACCTTCTTGAT
*Reg3*bg	GGCTTCATTCTTGTCCTCCA	TCCACCTCCATTGGGTTCT
*Reg3*g	AAGCTTCCTTCCTGTCCTCC	TCCACCTCTGTTGGGTTCAT
ubd	GCTTCTGTCCGCACCTGTGTTGT	CCTGCTTCACCACCTTCTTGAT

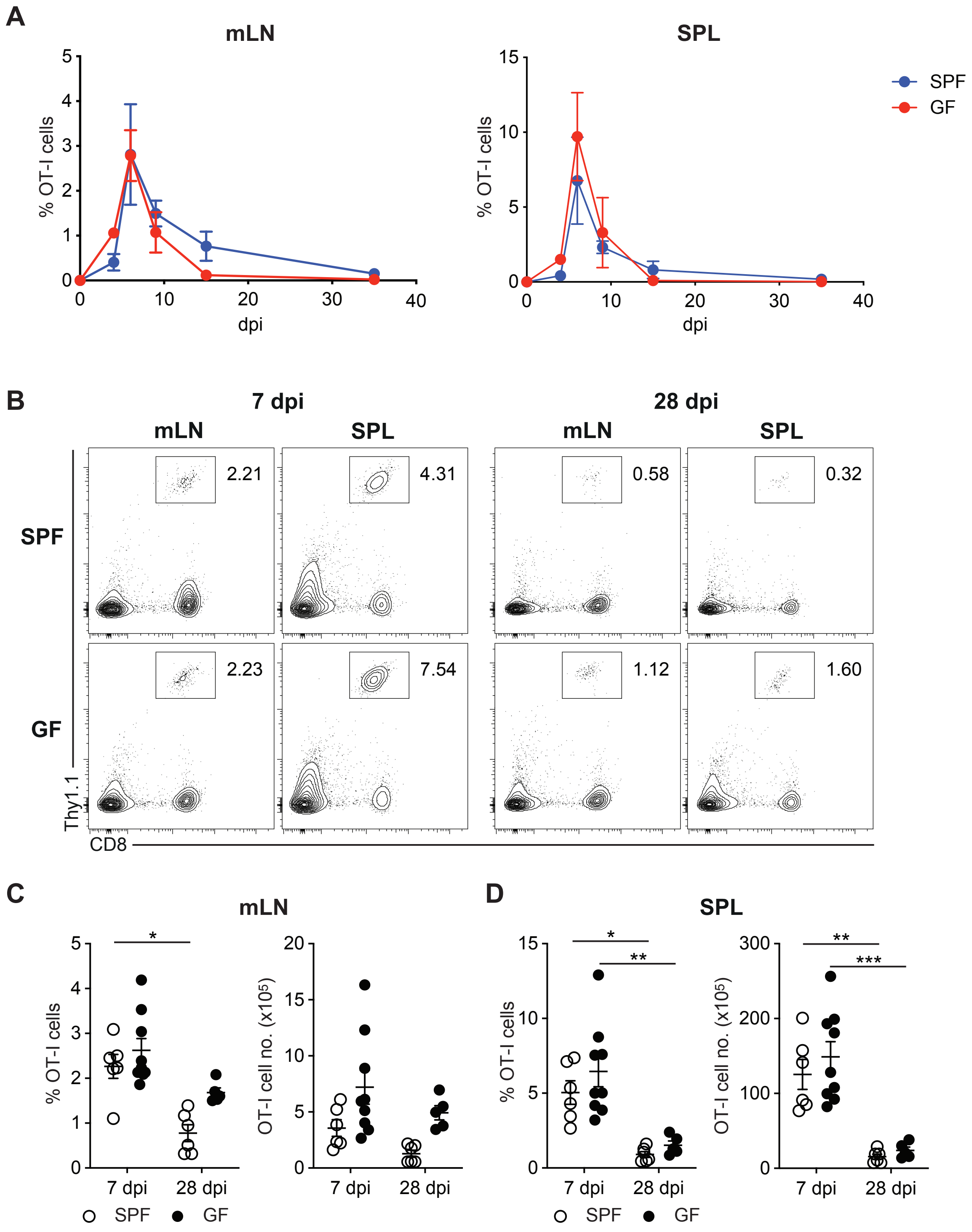

Supplementary Figure 1. Recovery of Donor OT-I cells in mLN and SPL During the Oral Infection of InlA^m *LM-OVA*. As shown in Figure 2, 5 x 10^5 OT-I cells were adoptively transferred into SPF and GF mice. Next day, mice were orally infected with InlA^m *LM-OVA*. (A) Percentage of donor OT-I cells gated on lymphocytes in mLN and SPL was determined at indicated time points (n=3 per each time point). (B-D) Data from repeated experiment. (B) Representative FACS plots showing the percentage of donor OT-I cells gated on lymphocytes. Percentage of donor OT-I cells (left) and total number of donor OT-I cells (right) in mLN (C) and SPL (D) at 7 and 28 dpi. Data are pooled from two independent experiments (n=6 for SPF 7 dpi, n=9 for SPF 28 dpi, n=9 for GF 7 dpi, n=5 for GF 28 dpi). Statistical differences were determined by two-way ANOVA with Bonferroni multiple comparisons. *P < 0.05, **P < 0.01, ***<0.001.

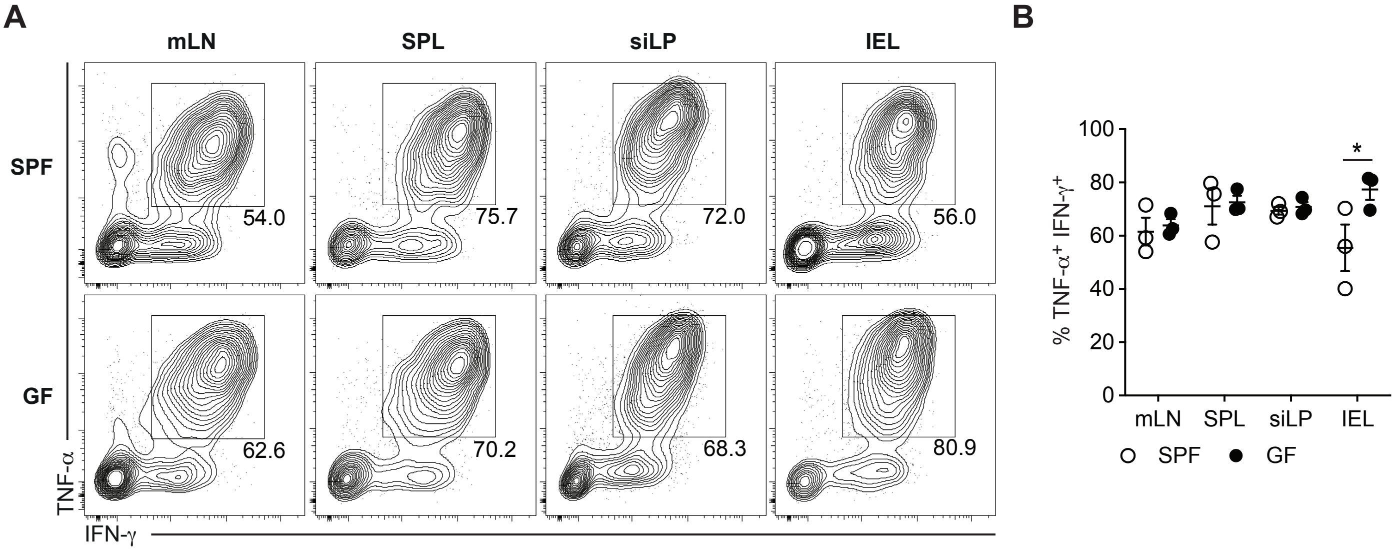

Supplementary Figure 2. Effector Functions of Donor OT-I cells in SPF and GF mice Orally Infected with InlA^m *LM-OVA*. 5 x 10^5 OT-I cells were adoptively transferred into SPF and GF mice. Next day, mice were orally infected with InlA^m *LM-OVA*. Single cell suspension from indicated tissues was prepared from SPF and GF mice at 7 dpi and was stimulated with SIINFEKL peptide for 4 hours. (A) Representative FACS plot showing T cells producing interferon-γ (IFN-γ) and tumor necrosis factor-α (TNF-α) gated on donor OT-I cells. (B) Frequency of IFN-γ^+ and TNF-γ^+ T cells among OT-I cells in indicated tissues from SPF and GF mice (n=3 per group). Statistical differences were determined by unpaired two-tailed Student's t test. *P < 0.05.

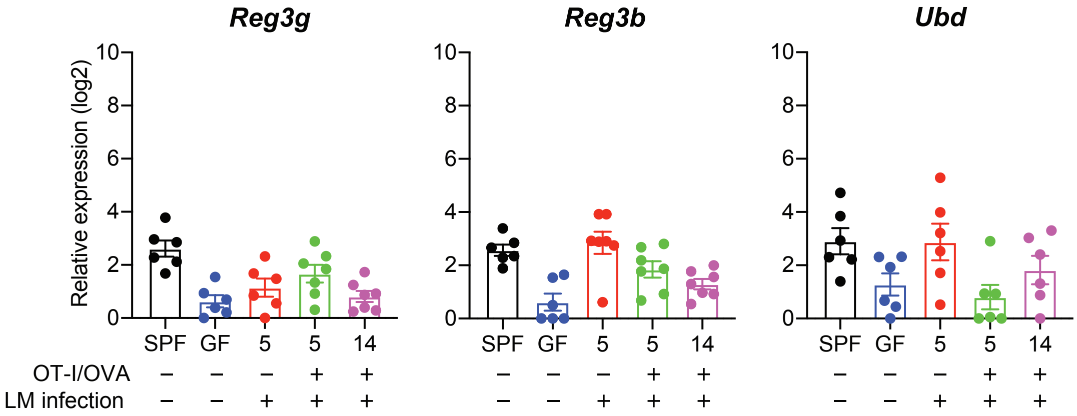

Supplementary Figure 3. Expression of Reg3g, Reg3b and Ubd in IECs from OT-I-Repopulated GF *Rag1*$^{-/-}$ mice During Oral Infection of *L. monocytogenes*. Total RNA were prepared from IECs of SPF and GF *Rag1*$^{-/-}$ mice, and OT-I-repopulated GF *Rag1*$^{-/-}$ mice at indicated time points during the oral infection of InlA^{m} *LM-OVA*. (A) Expression of *Reg3g* (left), *Reg3b* (middle), and *Ubd* (right) was measured by qRT-PCR. Data are pooled from two independent experiments (n=6 per group).
